# Communal Load Sharing of Miscarriage Experiences: Thematic Analysis of Social Media Community Support

**DOI:** 10.2196/56680

**Published:** 2024-12-10

**Authors:** Julia Dubbelman, Jonelle Ooms, Laura Havgry, Lianne Simonse

**Affiliations:** 1 Department of Design, Organization, and Strategy Faculty of Industrial Design Engineering Delft University of Technology Delft Netherlands

**Keywords:** miscarriage, miscarriage grief, online health communities, thematic analysis, social support, communal load sharing, peer-to-peer support

## Abstract

**Background:**

Miscarriage is a common experience, affecting 15% of recognized pregnancies, but societal ignorance and taboos often downplay the mental distress and personal impact following a miscarriage. Emerging stories on social media in which women express their miscarriage grief are breaking such taboos. Research in the area of online health communities is increasingly focused on studying how people share their health experiences on social media. However, a clear understanding on the social support involved in this type of sharing of health experiences is lacking.

**Objective:**

This study explored the use of Instagram in sharing miscarriage experiences, guided by the following research question: How is social community support given to women who share their miscarriage experiences on social media? Considering that social media is increasingly used as a source of social support, in this study, we chose Instagram as the social media platform. The purpose of this research was to create a better understanding of how social media provides support in expressing personal miscarriage experiences and how people engage with such posts.

**Methods:**

This study used a qualitative inductive research method in which a phenomenological strategy and thematic analysis were followed to create a comprehensive understanding of the social community support phenomenon. The dataset was established from a sample of 258 Instagram posts and 736 comments collected over a period of 6 months after initial posts and from 6 different women. These data were categorized and clustered through a thematic analysis.

**Results:**

Three themes were identified: (1) storytelling of emotional turmoil and grief after miscarriage, (2) sharing positivity amidst miscarriage grief, and (3) mentioning personal medical information about miscarriage. Theme 1 represents the emotional experience of women who have had a miscarriage. It encompasses the initial posts that included miscarriage storytelling that express deep grief and mental distress and the emotional impact on both the posters and the commenters. Theme 2 highlights the importance of finding moments of joy and positivity in the midst of mental distress and pain. The posts shared with the online community convey a sense of moving forward and a refusal to let grief become the defining aspect of one’s life. Theme 3 focuses on sharing medical and practical advice. This theme includes posts and comments about medications, in vitro fertilization procedures, hospital experiences, and personal physical symptoms.

**Conclusions:**

As an overarching theme for this social support phenomenon, we introduce the term *communal load sharing* to describe the therapeutic role of social media in helping women cope with miscarriage by providing a platform for sharing similar experiences, breaking social taboos, and fostering *load sharing*.

## Introduction

### Social Relevance

Miscarriage is a more common phenomenon than people seem to realize, occurring in 15% of recognized pregnancies [1]. Miscarriage is defined by the European Society of Human Reproduction and Embryology as the loss of pregnancy before 22 weeks of gestation [1]. One in 4 women will experience a miscarriage during their lifetime [2], so why do so few women know how common it is and what effects a miscarriage can have on a person’s life? Past research has indicated that societal ignorance and taboos often overshadow the profound effect miscarriage has on a person [3]. Over half of the women who have a miscarriage will experience lasting psychological effects [1,4-6]. Overall, there is an imbalance in research, as the physical effects of a miscarriage are prioritized over other effects, such as social-psychological effects. This study investigates these social-psychological effects, concentrating on postmiscarriage interactions on social media. Exploring the experiences women have with sharing their stories of miscarriage on social media can facilitate an understanding of how social media platforms can be used as a form of social community support [7]. Collecting and analyzing multiple Instagram users’ experiences can yield a holistic overview of the miscarriage experience. Through the longitudinal analysis of social media use, this research aims to create a better understanding of the role that social media plays in offering social support following a miscarriage experience. Specifically, this research considers Instagram users who shared their miscarriage experiences. Instagram is utilized as a source of data because it is currently one of the biggest social media platforms with >2 billion monthly users [8]. Against the background of online health communities (OHCs) research, the use of social media is a recognized direction in which OHCs are headed in the provision of social community support [9].

### Structure of the Paper

This paper contributes by unpacking important themes for social support on social media OHCs and introduces the overarching term *communal load sharing* to describe the therapeutic role of social media in breaking social taboos and fostering the sharing of similar experiences, ultimately helping women to cope with miscarriage grief.

The subsequent section provides a review of the theoretical background regarding different factors influencing the experience of a miscarriage in relation to social support. This review leads to the research question, followed by the Methods section in which the steps of thematic analysis are described with respect to the sample and analysis of the OHC created by sharing miscarriage experiences on Instagram. This is followed by the Results section, which presents the key findings on identified themes, evidence quotes, and visuals. Finally, the theoretical and practical implications are discussed, including limitations and future research opportunities.

### Theoretical Background

#### Mental Distress After a Miscarriage

While the physical consequences of miscarriages are well known, the psychological impacts are often overlooked. Half of the women experiencing a miscarriage face psychological distress, including anxiety, depression, guilt, and shame [4,10]. Moreover, some women also struggle with feelings of failure or envy of others’ children, which can lead to isolation [11]. Furthermore, concerns about future pregnancies, including the fear of experiencing another miscarriage, are commonly reported [4]. In addition, it is important to recognize that the impact of miscarriage leads to an increased use of mental health services and prescription drugs [12]. These psychological consequences may not always be evident in social interactions with health care providers, family, and friends, as they can lack outward physical manifestations [1]. Although miscarriage is often perceived primarily as the woman’s experience, it is important to recognize the impact it has on her partner. Despite their own substantial grief, some women report that the support that is offered overlooks their partners [13]. Men tend to grieve less intensely and for shorter periods than women following a miscarriage and have been found to use different coping mechanisms [14]. These differences in response to a miscarriage may contribute to misunderstandings and conflicts in the relationship, and studies have shown that couples have an increased risk of separation following a miscarriage [11,12]. Individuals can often feel unsupported due to differences in outward physical manifestations and reactions to miscarriage [15]. The social sharing of individual experiences might benefit the postmiscarriage coping and recovery process and decrease mental distress. However, a clear understanding of such social sharing and support is currently lacking.

#### Social Taboo Surrounding Miscarriage

Discussing miscarriages can be a sensitive and complex topic because of the social taboo that surrounds it. This taboo not only makes it difficult for individuals and couples to find support but it also perpetuates harmful myths and misconceptions surrounding miscarriage [3]. A common cultural habit of waiting until the 12th week to announce a pregnancy can further contribute to the negative psychological effects in the event of a miscarriage [16]; if a miscarriage occurs before friends and family are informed, the grieving process may be more difficult. The social stigma also persists in professional settings [17], where women frequently lack recovery time following a miscarriage [12]. Overall, this highlights the importance of breaking down the social stigma and providing adequate social community support for women experiencing miscarriage. More open and supportive communication around this topic and the creation of supportive environments both online and offline might be beneficial. By participating in OHCs through social media, individuals and couples who have experienced a miscarriage might feel less alone and more understood, which ultimately can lead to positive effects on processing the loss [12]. That is why, in this study, we explore the social community support of those who break the social taboo and share their miscarriage experience on social media.

#### Social Support Following Miscarriage

Prior research has indicated that one of the crucial factors for helping individuals through the experience of a miscarriage is social support [4,6]. This type of support is often offered by the patient’s partner, health care professionals, relatives, or friends. Studies have found that a lack of social support leads to isolation from loved ones [1,11]. However, in recent years, there has been a shift in the way people seek health information and emotional support in OHCs [18,19]. Traditionally, OHCs were forums administered by medical foundations or companies, providing a certain level of reputable health information [20]. However, with the rapidly increasing popularity of social media, a form of OHCs started emerging from these platforms as well [21]. In this research, we study social media–based OHCs that have the same function of offering a comparatively safe space for people dealing with miscarriage to share their experiences, find coping strategies, reduce stigma, and share emotional support [22]. As social media is increasingly serving as a source of social community support, it is crucial for health care professionals to recognize its importance in facilitating access to social support for people affected by miscarriage [23]. Moreover, this is also the type of support that health care professionals currently do not provide, as it must be tailored to each individual’s needs [5]. Recent studies have found that talking about miscarriage, listening, and providing informative and emotional support through different social networks can improve the quality of care [3,12,24,25]. Two additional reasons for this improvement challenge could be that it is not completely understood how individuals react to miscarriages [5,26] and that health care professionals do not feel educated enough to provide the necessary postmiscarriage support [12]. In this research, we consider social media as an important medium for social community support. On Instagram, there are numerous accounts with posts about the topic of miscarriage. We also recognize that the act of posting about miscarriage on social media is already a form of breaking the stigma surrounding miscarriage. That is why our research explores the social community support for the mental distress that many women go through after a miscarriage.

Given the recognized gap in miscarriage support from friends, family, and health care professionals and the evolving knowledge base on OHCs and the social support offered by community members, we pose the following research question: *How is social community support given to women who share their miscarriage experiences on social media?*

## Methods

### Qualitative Research Design

Social media, as a form of social community support, is a relatively new area of research. Therefore, we chose to use a qualitative research approach to explore this area and create an in-depth understanding [27]. Within this qualitative research approach, we applied a phenomenological strategy [28,29] to create a comprehensive understanding of the social community support phenomenon. For this purpose, we collected Instagram posts from a selected group of women sharing their miscarriage stories to analyze their perceptions and social support interactions on experiencing a miscarriage on a longitudinal basis. This data collection method enabled us to provide insights and a clear overview of social community engagement in the use of Instagram when sharing stories about miscarriage and how other users engage with the posts. The data, consisting of posts and corresponding comments, were collected within a 6-month time frame following the first post announcing the miscarriage. These posts were not limited to those explicitly addressing the topic of miscarriage; all other posts were also considered relevant for the research, as they contributed to forming a comprehensive overview of an entire miscarriage journey. A 6-month time frame was chosen to narrow down the research dataset, although additional posts about miscarriage were made after this time frame. During data collection, all aspects of engagement were gathered, including posts, comments, and likes. The data were later analyzed using thematic analysis to identify themes and relate each theme to its dynamic over time. We used a thematic analysis to unpack the multifaceted dimensions of social community support for miscarriage experiences.

### Instagram as a Data Collection Platform

Due to their growing popularity, social media platforms such as Instagram have become valuable sources of data for researchers seeking to explore various aspects of social science [30]. This approach recognizes the reliability and value of the stories and experiences shared by individuals on social media and highlights the potential for using social media platforms as a source of data for qualitative research. Our research focuses on 2 aspects of using Instagram as a data collection platform: how users share their stories and how other users engage with these posts. Instagram is a popular social networking platform for photo and video sharing that offers a rich and varied multimodal dataset beyond just visual content. This diversity makes it an invaluable source of insights into user interactions, narratives, and community dynamics. Table 1 provides an overview of the data extracted from Instagram in this research and the relevant terminology used by Instagram [31].

**Table 1:** Multimodal data collected from Instagram.

Name	Definition
Post	A shared picture or other visual content. Either a single picture or a series of up to 10 visuals (referred to as a carrousel)
Post caption	The text below a picture. Has a limit of 2200 characters
Picture comments	Other Instagram users’ comments’ on the post
Video	A short video of a maximum of 90 seconds
Video caption	The text below a video. Has a limit of 2200 characters
Video comments	The comments on a video
Nested comments	The comments inside another comment
Number of likes on posts and videos	The total amount of likes from other Instagram users at the time of data extraction
Number of comments on posts and videos	The total amount of comments from other Instagram users at the time of data extraction
Number of resends on posts and videos	The total amount of times the post or video is forwarded from one Instagram user to another at the time of data extraction

### Sample

The sampling strategy was focused on identifying women who have experienced a miscarriage and shared their stories on Instagram. To build a relevant sample, this research considers miscarriage following pregnancy from both traditional intercourse and in vitro fertilization from heterosexual relationships. The inclusion and exclusion criteria are provided in Textbox 1.

**Inclusion and exclusion criteria.**

**Inclusion criteria**

- Miscarriage after both in vitro fertilization and traditional intercourse
- Accounts with 10,000-100,000 Instagram followers
- Women who have experienced a miscarriage 6 months to 5 years prior
- English language
- Accounts from North American and European women
- Women aged >18 years

**Exclusion criteria**

- Nonhetero relationships
- Motivational accounts
- Less than 10 posts explicitly mentioning the miscarriage
- More than 75 overall posts
- Inability to give informed consent

The research identified those Instagram users who are so-called “micro-influencers” (10,000-100,000 followers) [32]. These influencers typically nurture more personal and engaging community interactions, essential for sensitive discussions about miscarriage. Their audience’s trust and relatability toward them promise more authentic and deeper insights into the subject. In addition, their niche following provides a concentrated yet diverse range of experiences, ensuring a rich and focused dataset for nuanced qualitative analysis. Furthermore, microinfluencers have a tighter-knit social community than larger or smaller accounts [32], which is highly relevant to this research, as its focus is on the use of Instagram as a social community. To begin the sampling, the hashtags #ihadamiscarriage, #miscarriage, and #1in4 were searched on Instagram to identify a list of potential microinfluencers. Instagram profiles that primarily focused on posting motivational content, often featuring quotes, positive affirmations, and generalized encouragement messages, were excluded as they differ distinctively from personal story-driven profiles. Once a list of potential posters had been made, these Instagram users were contacted via direct message on Instagram and provided with an informed consent form with the topic and purpose of the study. Posters were only included in the study when they gave direct permission to use their data. The time frame for post inclusion was chosen to be between 6 months and 5 years ago. This period is long enough to accumulate a substantial body of data for analysis, and the upper limit of 5 years ensures that the collected data are still relevant and reflective of current social media trends and discussions surrounding miscarriage. Profiles with content in English were selected to minimize translation biases and miscommunication. North American and European women were selected, as their social media use habits are similar [33]. Finally, Instagram profiles with at least 10 posts specifically mentioning the miscarriage were included to have enough robust data to analyze posting on social media about miscarriage. From a potential list of 9 microinfluencers who consented to the use of their data for this research, the number of posts the person made within the 6-month time frame was used as a further exclusion criterion; any influencers with >75 posts in 6 months were excluded. This exclusion led to a more tightly focused analysis that delved deeper into the sampled dataset. This ultimately resulted in a sample of 6 female Instagram posters who discussed their miscarriage experiences, with a total of 258 posts, 736 comments, and 1734 quotations, as shown in Table 2.

**Table 2:** Sample of collected data.

Poster	Followers^a^, n×1000	Posts analyzed^b^, n (%)				Comments analyzed^b^ (n=736), n (%)		Quotations, (n=1724), n (%)
		Total (n=258)	Videos (n=88)	Pictures (n=170)				
1	19.3	52 (20)	27 (31)	55 (32)	149 (20)		283 (16)	
2	65.5	41 (16)	28 (32)	13 (8)	119 (16)		271 (16)	
3	27.3	29 (11)	4 (5)	25 (15)	74 (10)		258 (15)	
4	22.3	57 (22)	9 (10)	50 (29)	171 (23)		331 (19)	
5	22.2	24 (9)	20 (23)	4 (2)	62 (8)		174 (10)	
6	12.0	55 (21)	0 (0)	55 (32)	161 (22)		407 (24)	

^a^Date of extraction: June 18, 2023.

^b^Date of extraction: between May 15, 2023, and May 31, 2023.

### Data Analysis

All data types shown in Table 1 were analyzed over a 6-month time frame to create a comprehensive and holistic overview of how the women share their stories online in interaction with other social media users. The top 3 comments under each post were analyzed, as these comments had the most likes from other users, indicating that these comments were the most popular among the people interacting with the post. In total, this was between 84 and 180 comments per poster. The number of data quotations analyzed was 1724 (Table 2). The first 3 authors were involved in the thematic analysis of the data sample [34]. The data were then structured in a code tree created collaboratively by these 3 researchers. The fourth author provided feedback and suggestions on the draft reporting of the code trees. Throughout this analysis process, discussions took place among all 4 researchers to refine and validate the themes and patterns, enhancing the validity of the findings through triangulation [34]. Throughout the coding process, the emergence of new themes and patterns was continuously tracked to determine if data saturation was achieved. Saturation was considered achieved when no new themes or patterns were identified in the final round of coding, which led to the creation of the final code tree. This gave an overview of the identified themes and the patterns of the presence of these themes during the time span following the first post about miscarriage.

### Six Steps of Thematic Analysis

To analyze the phenomenon of social community support for women who share their miscarriage experiences on social media, a thematic analysis approach was applied to determine recurring themes and patterns within the data [28,29]. Both the posts and the comments on the posts were analyzed separately for themes. The six steps highlighted by Braun and Clarke [28] were used: (1) familiarizing with the data, (2) generating initial codes, (3) searching for themes as clustered codes, (4) reviewing the themes, (5) defining and naming the themes, and finally (6) producing the report. The first 2 steps were largely carried out during the data collection described in the Sample section.. This was followed by the initial analysis, which included steps 3 and 4, where the first round of codes was determined (72 codes). Clustered codes (25 codes) were made to include codes that were not used as frequently but which were still important for the research. Following this, steps 5 and 6 were completed to make categories for both the posters and the commenters and then define and name the final themes.

### Ethical Considerations

This study has carefully taken ethical considerations into account, given the sensitive nature of the topic. The researchers have ensured that the study only uses stories and data from public Instagram accounts, shared by the individuals themselves. To ensure the privacy of the posters, the research team obtained consent for data use from all analyzed users who have shared their personal miscarriage stories through an Instagram post. The consent included informing the users about the use of their data and the purpose of the study and ensuring that their personal information is not disclosed in any way that could potentially identify them. The data of users engaging in posts (through comments and likes) were also treated with discretion and anonymized for confidentiality to ensure their privacy rights are respected. The project received ethics approval from the Human Research Ethics Committee at Delft University of Technology (approval number 2234), ensuring that the study is conducted in an ethical and responsible manner. The researchers approached this study with sensitivity and respect for the experiences of women who have experienced a miscarriage, making a conscious effort to use language that is not stigmatizing or insensitive.

## Results

### Social Community Support for the Multifaceted Experience of Miscarriage

**Figure 1:**
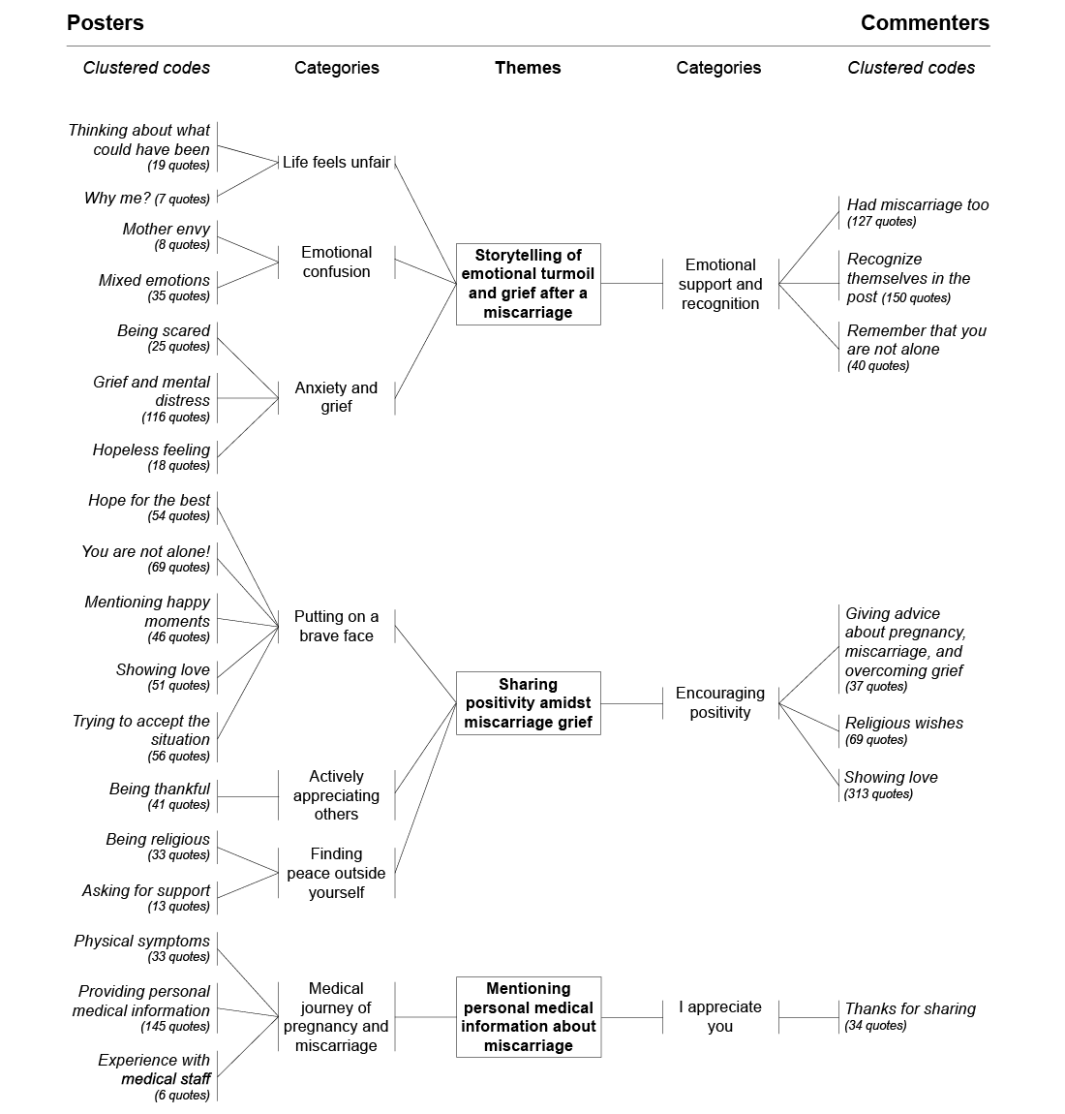
The dual code tree of social community support for miscarriage experiences.

Resulting from the thematic analysis, three core themes emerged: (1) *storytelling of emotional turmoil and grief after miscarriage,* (2) *sharing positivity amidst miscarriage grief,* and (3) *mentioning personal medical information about miscarriage.* These themes collectively represent how diverse experiences and emotions related to miscarriage are shared within the open social media community of Instagram. Figure 1 presents the dual code tree on social community support for miscarriage experiences. The 3 core theme structure results from the data induction of both the women posting their miscarriage experience (18 code clusters into 7 categories) and the online community commenters (6 code clusters into 3 categories).

Theme 1, *storytelling of emotional turmoil and grief after miscarriage,* uncovers the deep emotional impact and distress faced by women who have suffered a miscarriage and how, in reaction to their storytelling, commenters provide social-emotional support and recognition.

Theme 2, *sharing positivity amidst miscarriage grief*, emphasizes the resilience and determination of women to find moments of joy and hope despite the underlying grief and how commenters play a social support role in providing uplifting positive support to peers.

Theme 3, *mentioning personal medical information about miscarriage*, highlights the practical support aspects of experiencing a miscarriage, with the sharing of medical encounters and providing of medical information. The commenters express great appreciation for their openness in sharing such personal details.

While theme 1 is more focused on the internal emotional struggles, theme 2 demonstrates the desire to highlight positive aspects amidst miscarriage grief, and theme 3 takes a less emotional and more practical stance. Overall, these 3 themes illustrate the multifaceted nature of social community support surrounding grief and miscarriage on Instagram and demonstrate how social media is becoming its own support pillar when dealing with grief following miscarriage. The evidence on the core themes and categories is explained in more detail in the next paragraphs.

### Storytelling of Emotional Turmoil and Grief After Miscarriage

**Figure 2:**
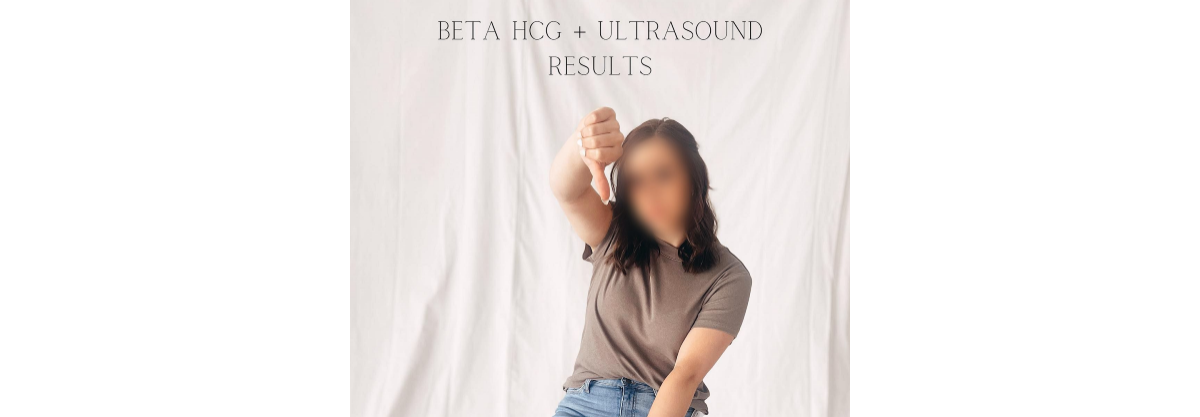
Evidence example of a posted image on theme 1.

The first identified theme is *storytelling of emotional turmoil and grief after miscarriage.* This theme represents the emotional experience of women who have had a miscarriage. It encompasses the initial posts of miscarriage storytelling that express deep grief and mental distress and the emotional impact on both the posters and the commenters (Figure 2). The posters express their grief by posting sincere pictures, heartfelt captions, and personal videos; sharing their story; or explaining the situation. The commenters often identify with these posts, and some of them open up about their own loss. Besides this expression of grief from both the posters and commenters, there is a strong element of encouragement that frequently accompanies these posts and comments. Three categories, in particular, were found to stand out among the posters: *life feels unfair*, *emotional confusion*, and *anxiety and grief*. For the commenters, the main category is *emotional support and recognition*.

In the first category of expressions, *life feels unfair*, the posters frequently mentioned how life seemed unfair and they questioned the reason behind their miscarriages (7 quotes), which also related to “feeling hopeless” (18 quotes). One community poster states the following:

So while I have reached a point of understanding and divine peace, we will all still grieve what could have been.

Poster 5

The second category, *emotional confusion* included several instances of mixed emotions (35 quotes). Some women felt envy toward other women who had successful pregnancies (8 quotes). One woman posted about having feelings that were difficult to navigate:

It’s been hard to see other pregnant people and be triggered with new announcements because I thought for sure our time would’ve come already.

Poster 2

The third category was about feeling *anxiety and grief*, pain, and mental distress. These emotions can be difficult to navigate, which can lead women to feel scared (25 quotes) and frequently, with women explicitly expressing grief in these posts (116 quotes):

I had never really felt despair in life before we lost you. There truly isn’t a day that goes by that I don’t think about you and wish you were here. and what makes it even harder is that our journey has brought even more heartbreak since then.

Poster 2

*Emotional support and recognition* was the category of community commenters who recognized themselves in the posted story and shared their recognition (150 quotes) or their own miscarriage story (127 quotes) and offered social-emotional support to the poster by expressing that they were not alone (40 quotes) with reactions such as the following:

Hugs mama, please know you are not alone. Thank you for your vulnerability.

Comment on poster 5

### Sharing Positivity Amidst Miscarriage Grief

**Figure 3:**
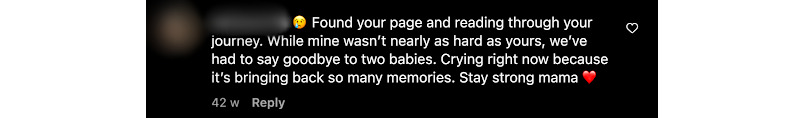
Evidence example of a comment related to theme 1.

This theme focuses on follow-up posts that differ from the initial ones in that they showcase moments of happiness and positivity, despite the underlying grief. These posts shared with the online community convey a sense of moving forward and a refusal to let grief become the defining aspect of one’s life. Each category—*putting on a brave face*, *actively appreciating others*, and *finding peace outside yourself*—served as a means of giving social community support back to those who might be dealing with a similar experience, thereby sharing the knowledge that even in the face of misery, there can be moments of joy. Within this theme, the main category for commenters is *encouraging positivity*, emphasizing the profound impact of cheerful and positive messages shared with each other, especially during the most challenging times (Figure 3). Commenter messages foster a sense of community support, expressing hope and encouragement for the poster.

The category *putting on a brave face* expresses trying to be upbeat even through the darkest of times. It encompasses various clustered codes such as being hopeful, *hope for the best* (54 quotes), spreading awareness about miscarriages, and making social community support explicit, coded as the expression *you’re not alone!* (69 quotes) to everyone in the online community, such as in the following quote:

If 2022 didn’t turn out to be your year, I just want to say that I see you. You’re not alone.

Poster 4

In addition to that, *putting on a brave face* involves the *mentioning of happy moments* (46 quotes), *showing love* (51 quotes), and the sharing of *trying to accept the situation* (56 quotes) amidst grief. For an example, see the shared photo shown in Figure 3 and the following quote:

I know one day I’ll look back at this time in my life and be proud of the woman I’ve become. I know it isn’t all bad. But grief is an interesting thing. It comes and goes, and in many ways stays with you forever. I know it also makes me appreciate aspects of my life more now...It isn’t all bad.

Poster 4

The next category of *being thankful* (41 quotes) concerned gratitude for the community support. Both commenters and posters showed gratitude for their partner in difficult times, and they also expressed their gratitude to the team of health care professionals, their loved ones, and community peers. For example, 1 woman wrote in the caption of a post asking for funding for their surrogate:

We are so grateful for the support we have gotten from you all and feel as though we can’t fully express how much we love you and appreciate your help and the love you send our way. Someday we will have a happy ending and we appreciate your help to get there.

Poster 1

Then another category under this theme concerns *finding peace outside yourself*, which refers to seeking meaning and reaching out for social community support by *asking for support* (13 quotes) and embracing religious beliefs, *being religious* (33 quotes), to provide emotional strength:

So when you cry out, in desperation, like I’ve done countless times, and feel like He isn’t opening the doors you want, or isn’t moving you in a certain direction, know that BEING STILL is sometimes the hardest, yet most important thing you can do. It is in these moments that we truly develop FAITH. That we give over our will to His and TRUST HIM. It’s when we progress beyond measure.

Poster 5

The main category for commenters was *encouraging positivity* by being supportive and *giving advice about pregnancy, miscarriage and overcoming grief* (37 quotes) to peers who struggled with the same feelings, with one commentator stating the following:

Glad y’all had a nice break together. We just took a break for a vacation as well! I can’t recommend it enough.

Comment on poster 4

Some commenters expressed their support by offering *religious wishes* (69 quotes), thereby showing solidarity and providing comfort:

I feel your pain. I had to pour into something to get me through. I was in a sad, lonely dark place.... no one can understand... until they have lived it. I choose to listen to Christian contemporary music, which helped and I leaned in and listened to many online service through our church app. We had many obstacles knock us down, but kept moving forward with new goals to make our family. We finally had twins! Luck and strength to you.

Comment on poster 1

Other commenters expressed empathy by *showing love*, exemplified with heart emojis and quotes such as the following:

Words cannot even express how sorry I am for your loss. Your post resonated with me so much. This pain is the hardest. I love you my friend. Sending you and [name of husband] all my love and thoughts during this difficult time.

Comment on poster 4

### Mentioning Personal Medical Information About Miscarriage

**Figure 4:**
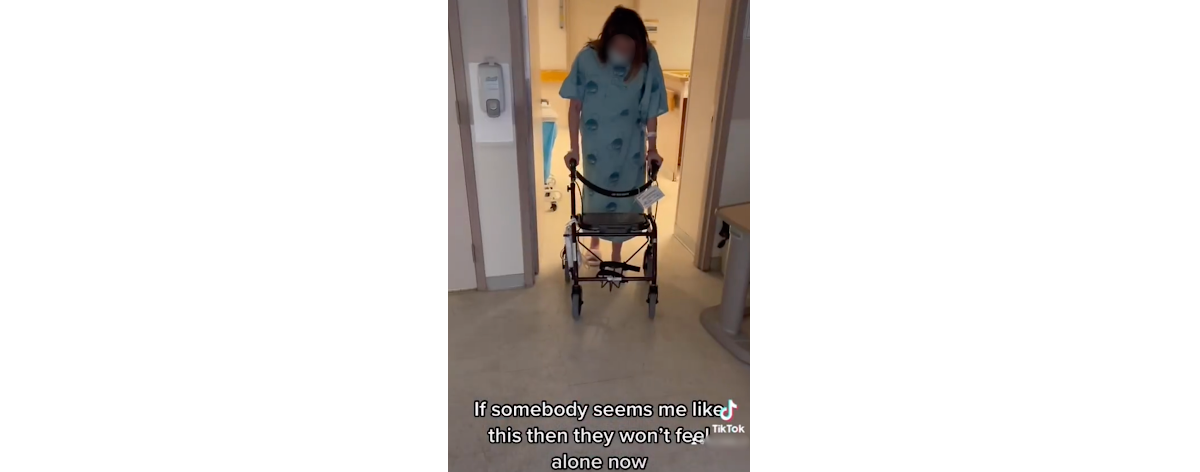
Evidence example of a posted reel related to theme 3.

The third theme related to providing practical information and support to peers who have experienced similar situations (Figure 4). It included posts and comments about medications, in vitro fertilization procedures, and hospital experiences. *Physical symptoms* were also shared, and personal advice was given and shared.

Connected under this theme was the category *I appreciate you* from commenters showing gratitude for sharing this information.

*Personal medical information providing* concerns the category of *medical journey of pregnancy and miscarriage*, which often is not an easy journey. As many posters shared, this involved taking pills, managing diets, making regular visits to a clinic, and various other actions, coded as *sharing personal medical information* (145 quotes). For example, 1 poster wrote the following:

So today until trigger I’ll be taking Omni trope—a human growth hormone—shot daily. 2.5mg to be exact, along with testosterone gel (1 pump) and DHEA 75mg (daily).

Poster 4

*Physical symptoms* (33 quotes) cluster codes of posts about sharing physical symptoms and side effects of treatments and medical procedures. This also included posts on appreciating the capabilities of the body and addressing difficulties with trusting their body after the miscarriage:

A constellation of battle scars. I once had someone refer to their scars as such and it has stuck with me ever since. I’ve been saying this for years, I truly believe that polar opposite feelings can co-exist. When I look down at my stomach, I’m torn between feeling really sad and also being proud of all that it’s been through.

Poster 4

Some also shared their personal encounters with health care professionals, encompassing both positive and negative experiences, coded as *experience with medical staff* (6 quotes):

And if I haven’t said it enough I really love my clinic and my medical team. My nurse called me today and just made me feel so seen and taken care of. They truly want this for us as well and are fighting with us and I just appreciate them so much. Having my Dr be so personally involved rather than just seeing a tech every time I go in also makes me feel so taken care of. I’m just very grateful for the team we have helping us navigate all of this.

Poster 4

Most commenters expressed their appreciation for the poster’s courage in openly sharing their story about the sensitive topic of miscarriages, making people who have gone through similar experiences feel less alone, coded as *thanks for sharing* (34 quotes):

Thank you for always being so open about it all! It helps feel less alone through it all.

Comment on poster 4

## Discussion

### Principal Findings

This research sought to better understand how women who have experienced miscarriage share their stories on social media and how commenters engage with these posts as a form of social community support. Its in-depth qualitative results offer, besides new insights into the longitudinal experiences of miscarriage grief and breaking down the stigma surrounding miscarriage through the use of social media, a novel contribution to the area of OHCs. In addition to the theoretical understanding of these domains, we identified three overarching themes that address the multifaceted social sharing of grief experiences after a miscarriage on social media: (1) storytelling of emotional turmoil and grief after miscarriage, (2) sharing positivity amidst miscarriage grief, and (3) mentioning personal medical information about miscarriage.

### Social Media Community Support

All 3 themes demonstrate how breaking the social taboo by sharing miscarriage experiences on social media can generate social community support in a reciprocal way. Breaking the taboo and stigma surrounding miscarriage is important to create an environment where women feel safe and supported in their healing process. In contrast to the research that indicated that the topic of miscarriage is too sensitive and complex to talk about [3], we found that social media lowers this threshold. Each time a story is shared by uploading a post, a conversation about miscarriage is started. This raises awareness about an often misunderstood topic and helps normalize grief after a miscarriage. Both posters and commenters are, perhaps unknowingly, challenging societal norms, and their actions contribute to fostering a better environment for those who have experienced this loss. The public sharing of miscarriage grief contributes to breaking down harmful myths and misconceptions about this topic. The 3 identified themes enhance an understanding of how social media can be used as a form of social support. This also relates to the theoretical implications for resolving an overall lack of knowledge on practical aspects of dealing with miscarriage grief [23]. It adds to previous research on social media that indicated that women often search for social forms of support on their own [23,30] but contradicts the research that suggests that the information shared is not valuable [35]. In particular, theme 3, *mentioning personal medical information about miscarriage*, indicates that the opposite is true: social media serves as a medium to educate others. Regarding this theme, the researchers consider it crucial to address the potential risks associated with medical information given by nonprofessionals. The information shared is based on personal experiences and may be inaccurate, as most of the posters and commenters lack the expertise to provide medical advice. This can lead to harmful consequences if others follow such advice without consulting health care professionals. While sharing personal medical information is perceived as helpful, as our findings show, relying solely on this information holds potential risks. Instagram users should seek professional medical help for health concerns.

### Multifaceted Miscarriage Grief

The themes *storytelling of emotional turmoil and grief after miscarriage* and *sharing positivity amidst miscarriage grief* are interconnected, as both have to do with emotions related to miscarriage grief. The first largely points to the negative emotional experiences following the miscarriage, while the latter shows how women are trying to cope with their grief and learning to accept the pain by actively focusing on the good things in life.

In this study, it has become evident that women experience profound grief following a miscarriage. The vast majority of the analyzed posts show aspects of an intense grieving process. The main finding of this study, evident in both themes, is the importance of not becoming consumed by grief and sadness. Instead, it highlights the value of actively seeking social support and, alongside sharing emotional struggles, also expressing positivity and gratitude during times of grief. The posters mostly receive uplifting comments and advice, fostering a positive environment of social media support for both the poster and the commenter. This corresponds to a recent study that indicated that social media provides a form of nurturing support, which focuses on consolation more than problem-solving [26].

This supplements the understanding of mental distress caused by a miscarriage. It identifies the multifaceted nature of miscarriage grief and sheds light on an important aspect of social media community support, that is, empathy and consolation from emotional recognition.

### Communal Load Sharing

All themes provide new insights into the reciprocal nature of social media community support. Both women who initiate personal storytelling on their miscarriage grief by posting on social media and their peers, the women who interact with these stories, are seeking consolation and empathy through sharing their emotional turmoil; medical information; and, unexpectedly, also their positivity in coping with their mental burden and load within the online community. This *communal load sharing* can be seen as a new form of social support, sought mostly through interactions on social media. Distinct from providing social support, either emotional or information support within OHCs, communal load sharing is a reciprocal sharing of grief experiences, positive moments, and social appreciation. Given the absence of a specific construct to describe this, we propose to use the term *communal load sharing* for this unique form of social support. Communal load sharing stands in contrast to prior studies that report on the indifference and lack of support from friends and family [1] and the consequences of self-isolation [11]. The novel finding in addition to existing research is the prominent outward manifestations of miscarriage experiences. Communal load sharing encapsulates this through social self-expression. The findings of the first theme, *storytelling of emotional turmoil and grief after miscarriage*, correspond with recent studies that indicated that women can have difficulty expressing their emotions following a miscarriage, although this is an integral step in the healing process [23,26]. Posting on social media about the miscarriage is a form of self-expression that can have positive effects on the healing process, and in this study, it was found to be a form of social self-expression in which women collectively cope with their miscarriage grief. This social self-expression and openness confront the often-understated depth of the experience.

In response, the social support from the commenters comes in the form of *emotional recognition support*, with commenters sharing their own stories and reminding the poster that they are not alone in their grief journey. This is in line with an earlier study in which women indicated that 1 of the most important factors that helped them following a miscarriage was hearing other women’s stories [4]. In the second theme, *sharing positivity amidst miscarriage grief*, the posters shared positive moments in contrast to the traumatic event of a miscarriage. The commenters appreciated these forms of positivity and reinforced them by expressing their happiness for the poster, wishing them well, and providing religious blessings.

The 2 forms of communal load sharing, *social self-expression* and *emotional recognition support*, also add to the theoretical understanding of OHCs [20,21]. This builds upon corresponding research in which social media has been found to not have the informational reputability and reliability that OHCs formed by medical foundations or companies have [22], although it serves as an increasingly frequently used source of online community support [18] and, as our research indicates, an important form of social community support.

### Limitations and Future Research

The researchers acknowledge the potential biases that may arise from using only publicly available data and recognize that it may not represent the full range of experiences with miscarriage and the perspectives of women who are not active on social media. As is inherent to every qualitative study, we acknowledge the specific limitations of this type of research owing to potential researcher bias when interpreting and analyzing data. This research was purposefully focused on creating a better understanding from a woman’s perspective; their partner also plays a large role in the grieving process following a miscarriage, and it would be worthwhile to conduct a study on the partner’s point of view in the future.

The thematic analysis was based on a large longitudinal sample of >250 posts and approximately 730 comments; however, the sample was small, comprising 6 Instagram profiles. This may limit the diversity of experiences and perspectives in the context of social support for miscarriages. It is worth noting that the clustered commenter code *showing love* had a significantly high presence. It would be an interesting subject for future research to look into the different ways of showing love. Furthermore, in addition to analyzing the highest engagement comments, additional research could study all types of comments, including low engagement posts; the prioritization of comments; and, if possible, any removed comments. While this research focuses solely on studying posts and comments, future research could extend this analysis to include comments on comments. Moreover, interviews with posters could also lead to a deeper understanding of the topic.

This study sampled data from microinfluencers on Instagram. Microinfluencers often have a more engaged audience in comparison to everyday users or mega-influencers. This engagement can result in more impact of the posts and responses. In addition, the characteristics of microinfluencers may also affect the type of interaction they attract. For further research, it would be interesting to explore the interactions of everyday Instagram users or mega-influencers to research how social community support is given on these accounts. This could provide a broader perspective on the role social media plays in communal load sharing.

### Practical Implications

The practical implications of these findings suggest that social media not only helps break the silence around the sensitive topic of miscarriage but also emphasizes the importance of cultivating a supportive online community, which can foster healing and understanding. Our research suggests that social media can be useful for providing social community support for women experiencing miscarriage and how they deal with grief. The main findings from this paper offer practitioners insights into how women navigate and cope with multifaceted miscarriage grief. All 3 themes demonstrate how communal load sharing is integral to social support for dealing with grief following a miscarriage. This encapsulates the interactions between posters and commenters through social self-expressions and emotional recognition support. Understanding women’s experiences of miscarriage enables the social support network to provide further targeted assistance. As mentioned previously, health care professionals often do not feel adequately trained to deal with miscarriages [4]; thus, this in-depth analysis of social media support communities can help medical professionals better understand their own role in supporting the patient.

The researchers hope that this paper can serve as inspiration to continue breaking the taboo around the topic of miscarriage and contribute to paving the way for a more empathetic and inclusive society, where the emotional well-being of those affected by miscarriage is acknowledged, valued, and supported.

### Conclusions

This study identified three themes that address the social sharing of grief experiences after a miscarriage on social media: (1) storytelling of emotional turmoil and grief after miscarriage, (2) sharing positivity amidst miscarriage grief, and (3) mentioning personal medical information about miscarriage.

As the overarching theme for this social support phenomenon, this study describes *communal load sharing* as a therapeutic role of social media in helping women cope with miscarriage by providing a platform for sharing similar experiences, breaking social taboos, and fostering load sharing.

## Abbreviations

OHC: online health community
